# Personality change in the Nottingham Study of Neurotic Disorder: 30-Year cohort study

**DOI:** 10.1177/00048674211025624

**Published:** 2021-07-10

**Authors:** Min Yang, Helen Tyrer, Tony Johnson, Peter Tyrer

**Affiliations:** 1 Swinburne University of Technology, Hawthorn, VIC, Australia; 2 Imperial College London, London, UK; 3 University College London, London, UK; 4 Medical Research Council Clinical Trials Unit, University College, London, UK

**Keywords:** Personality disorder, change, follow-up, stability

## Abstract

**Background:**

Persistence is said to be a feature of personality disorder, but there are few long-term prospective studies of the condition. A total of 200 patients with anxiety and depressive disorders involved in a randomised controlled trial initiated in 1983 had full personality status assessed at baseline. We repeated assessment of personality status on three subsequent occasions over 30 years.

**Methods:**

Personality status was recorded using methods derived from the Personality Assessment Schedule, which has algorithms for allocating Diagnostic and Statistical Manual of Mental Disorders (DSM) and the 11th International Classification of Diseases (ICD-11) categories. The category and severity of personality diagnosis were recorded at baseline in the randomised patients with DSM-III anxiety and depressive diagnoses. The same methods of assessing personality status was repeated at 2, 12 and 30 years after baseline.

**Results:**

Using the ICD-11 system, 47% of patients, mainly those with no personality disturbance at baseline, retained their personality status; of the others 16.8% improved and 20.4% worsened to more severe disorder. In DSM-III diagnosed patients, those diagnosed as Cluster A and Cluster C increased in frequency (from 14% to 40%, p < 0.001, and 21.5% to 36%, p < 0.001, respectively) over follow-up, while those with Cluster B showed little change in frequency (22% to 18%, p = 0.197).

**Conclusion:**

In this population of patients with common mental disorders, personality status showed many changes over time, inconsistent with the view that personality disorder is a persistent or stable condition. The increase in diagnoses within the Cluster A and C groups suggests personality disorder generally increases in frequency as people age.

## Introduction

Personality traits tend to be enduring (Costa and McCrae, 2002). It has been therefore assumed for many years that personality disorder is equally persistent and so adjectives such as ‘pervasive’, ‘‘ingrained’ and ‘enduring’ have been part of the diagnostic description of personality disorder in both *Diagnostic and Statistical Manual of Mental Disorders* (DSM) and International Classification of Diseases (ICD) for over 50 years. But there has been much evidence to show that personality disorder, as opposed to personality traits, is not a stable diagnosis.

Several longitudinal studies (Lenzenweger et al., 2004; McGlashan et al., 2005; Zanarini et al., 2003) have demonstrated that personality disorder, using current diagnostic rules, changes greatly over periods of 2–6 years, mainly towards improvement to no personality disorder. In some respects, this is to be expected, as the diagnostic criteria include episodes of symptoms and behaviour that do not necessarily persist. It is fair to add that studies of self-reported personality assessments in the form of dimensional traits show much greater consistency than interview assessments (Morey et al., 2012), particularly on one measure, the Schedule for Normal and Abnormal Personality (SNAP), that integrates both normal and abnormal personality traits (Clark et al., 2014). There is also evidence that over a 2-year period that changes in personality traits are reasonably accurate predictors of change in personality disorder subsequently, but this was not shown with obsessive-compulsive disorder (Warner et al., 2004).

There have been no studies in which personality change has been assessed repeatedly over a very long period apart from the McLean study headed by Zanarini and her colleagues. This has involved repeated assessments in 275 patients with borderline personality disorder at 4 yearly intervals with good follow-up to 24 years. However, this was a highly unusual population as all the patients recruited were inpatients with borderline personality disorder, and a large number of treatments were given for the disorder over the course of follow-up (Zanarini et al., 2015). Zanarini et al. also included a comparison group of other mixed personality disorders in their studies. In both the Zanarini and larger Collaborative Longitudinal Personality Disorders Study (CLPS), avoidant personality disorder had the best outcome of all personality disorder categories (Grilo et al., 2004; Gunderson et al., 2011).

But these studies have all been in patients assessed *and* treated for personality disorder and so constitute a selected sample. In the Nottingham Study of Neurotic Disorder (NSND), personality status was assessed as an additional measure in a study of anxious and depressed patients presenting at general practice psychiatric clinics (meaning that they were seen at an earlier stage in management than those presenting as outpatients) (Tyrer, 1984). This cohort therefore included patients both with and without personality disorders at baseline. Because there was considerable uncertainty over the course of personality disorder at the time the study was initiated in 1983, the opportunity to repeat assessments was made after baseline at 2, 12 and 30 years by assessors ignorant of initial personality status.

Mortality in the Nottingham Study and clinical status up to 30 years have been reported elsewhere (Tyrer et al., 2021a, 2021b).

## Method

The patients were recruited from psychiatric clinics in eight general practice surgeries in Nottingham between 1983 and 1987. Such clinics were widely used in the area in the 1980s (Tyrer, 1984, 1989). The patients were originally entered into a randomised trial carried out over 10 weeks, in which, using constrained randomisation, 210 patients were allocated to drug treatment (*n* = 84) (the antidepressant, dothiepin [*n* = 28], the anti-anxiety drug, diazepam [*n* = 28] and placebo [*n* = 28]); cognitive behaviour therapy (*n* = 84); and self-help (*n* = 42). The results of the trial have been described previously (Tyrer et al., 1988b, 1990). Personality status had no influence on outcome over this short period.

Ethical approval for follow-up was granted by Northampton Research Ethics Committee (12/EM/0331).

### Assessment of personality

Personality assessment was made at baseline by previously trained independent researchers (all psychiatrists) using the Personality Assessment Schedule (PAS) (Tyrer and Alexander, 1979; Tyrer et al., 1979). This is an interview schedule carried out by a trained observer taking about 45–60 minutes to complete and assesses 24 personality attributes, each on an 8-point scale. The scores are subsequently classified into two groups, one to assess severity and the other to assess the type of personality disturbance. The PAS is a combined categorical and dimensional scale similar to the SNAP (Clark et al., 2014).

The categorical diagnoses from the PAS are grouped into four on the basis of a previous factor and cluster analysis (Tyrer and Alexander, 1979) as antisocial, dependent (but subsequently passive-dependent), inhibited (later anankastic) and withdrawn (later schizoid) (Tyrer et al., 1990). The ICD-11 severity levels (Tyrer et al., 2019), including the sub-syndromal condition, personality difficulty, were included retrospectively after a previous analysis (Tyrer et al., 2014).

The categorical groups of personality from the *Diagnostic and Statistical Manual of Mental Disorders* (3rd ed.; DSM-III) classification were also recorded at baseline using a separate algorithm derived from the individual items of the PAS (Tyrer et al., 1988a: 166–167).

Personality status was assessed again in the same way as at baseline at 2, 12 and 30 years, on each occasion by face-to-face contact and with observer-rated interviews. The initial (baseline) assessors carried out the assessments at 2 years, while H.T. assessed most of the patients at 12 and 30 years, unaware of both initial diagnosis and all other information at baseline.

The main hypothesis at the start of the study was that personality status would remain essentially stable over time. We examined this in three different ways:

1. Calculating the proportion of subjects with each personality disorder type at each of the four time points using both PAS and DSM-III systems;
2. Examining the changes in the proportions of subjects over time, by no change, improvement to no personality disorder, worsening to more personality disorder or fluctuating over time;
3. Comparing changes in each of the three commonly described clusters of personality disorder, A, B and C, originally suggested in DSM-III (American Psychiatric Association, 1980: 307).

Severity of personality dysfunction at each time point was recorded at an individual status level from baseline diagnosis over the follow-up period.

## Statistical methods and analysis

For the proportions of personality disturbance over the four time points, we first used the chi-square statistic to test the linear-to-linear test for change in the marginal distribution of subjects with personality disorder over time. For patients with 2, 3 and 4 measures of personality status, we defined a variable of four categories to reflect change in personality type at the individual level from the first time point to the last one: no change, improved (from any personality disorder to no disorder, or from more personality disorder types to less), worsened (from none to any personality disorder or from less to more types of disorder) or fluctuators (change from none to any, then back to none, or vice versa; change from one personality type to more, then back to less, or vice versa). The distribution of the change category between time points was compared using the chi-square statistic to test consistency in the change pattern.

Given the nature of repeated measures with missing data at different time points and known comorbidity of personality groups, as well as binary outcome of personality disturbance, we used multilevel multivariate logistic models (Yang and Goldstein, 2000). These allowed us to include most patients (*N* = 207) in estimating change trends of all PD types jointly with full variance-covariance structure of the PD positive rates at both individual and measurement levels, for maximum statistical efficiency. In models, follow-up time was treated as a continuous variable (0, 2, 15 and 30 years), which allowed us to test changes with time both linearly and nonlinearly.

The same modelling analysis was also used for examining changes in personality status by subgroup of those with different severity status at baseline, in particular those without any personality disturbance and those with personality difficulty.

We used SPSS v19 for descriptive analysis and MLwiN V2.3 for modelling analysis.

## Results

All patients recruited to the study had a DSM-III diagnosis of dysthymic, generalised anxiety or panic disorder, and those with both anxiety and depressive disorders were analysed separately as mixed mood disorders or cothymia (Tyrer et al., 2001).

Of 210 cohort participants, 165 had full measures at 2 years follow-up, 1 had died and 44 (21.0%) were lost to follow-up. At 12 years follow-up, some participants were assessed who did not have measures at 2 years (Tyrer et al., 2004). Seventeen (8.1%) had died, 185 had clinical assessment and 15 (7.1%) did not have PAS personality measures. At 30 years follow-up, 71 (33.8%) had died, 87 had personality assessment and 54 were lost to follow-up (25.7%). We compared characteristics at baseline, including age, gender, marital status, social class, initial treatment group, DSM diagnosis and general neurotic syndrome status of individuals who died, who were lost to follow-sup and who were assessed by 2, 12 and 30 years follow-up time, respectively, by means of both univariate and multivariate testing. The result (Supplementary Table S1) indicated that individuals who had died during the follow-up period were significantly older than others. Otherwise, all groups of individuals had similar characteristics during the study period. It suggested missing measures were at random, but adjustment for age might be made in further modelling analysis. This was done in the analysis of personality change at the individual level.

In total, 200 patients had a complete assessment of personality status at baseline. Of these, 11 (5.3%) had only one measure of personality status, 31 (15.0%) had two, 95 (45.9%) had three and 70 (33.8%) had four measures. Seventy-one (33.8%) of the cohort had died over the 30-year period, and 50 (23.8%) were lost during follow-up, approximately one half because of difficulties in contacting them, and the remainder from refusals to be seen.

The availability of data by follow-up time and personality disorder status (absent/present) for both ICD and DSM is shown in the Supplementary Table S2.

### Changes in nature of personality disorder over follow-up

At baseline, 73 (36.5%) of the 200 patients had at least one personality disorder (PAS system) and 78 (39.0%) (using DSM-III), but as there was comorbidity of disorders the numbers of named disorders were 84 in the PAS system and 115 in the DSM-III Clusters. The proportions of subjects with a personality disorder decreased over time for antisocial and histrionic personality disorders but for others in the Cluster B group (borderline and narcissistic) there was little change. However, Cluster A (schizoid, paranoid, avoidant and schizotypal) and Cluster C (anankastic, passive-dependent) personality disorders increased over time (Table 1). The number of patients assessed at 30 years was fewer, mainly because of death, but examination of those who had died at earlier time points showed similar proportions of personality disorder to those who were assessed in person (Tyrer et al., 2021a).

**Table 1.** PAS and DSM personality type over time and tested by repeated-measures model at patient level.

PD type	Positive cases and percentage (%) at follow-up time points					Model estimated change parameter	
	Baseline *N* = 200	2 years *N* = 162	12 years *N* = 186	30 years *N* = 89	χ^2^ (*p*) for linear trend	Linear change AOR [95% CI]^a^	Quadratic change Est (SE)
PD type (PAS system): *n* (%)							
Sociopathic	27 (13.5)	8 (4.9)	9 (4.8)	8 (9.0)	4.50 (0.034)	0.988 [0.965, 1.012]	0.0047 (0.0017)**
Passive dependent	27 (13.5)	14 (8.6)	29 (15.6)	20 (22.5)	4.19 (0.041)	1.027 [1.008, 1.047]**	−0.0000 (0.0011)
Anankastic	21 (10.5)	13 (8.0)	27 (12.7)	17 (19.1)	5.27 (0.022)	1.028 [1.007, 1.049]**	−0.0009 (0.0011)
Schizoid	9 (4.5)	10 (6.2)	31 (16.7)	14 (15.7)	17.74 (0.000)	1.041 [1.017, 1.066]***	−0.0043 (0.0012)**
** **Any PD	59 (29.5)	32 (19.8)	60 (32.2)	34 (38.2)	3.16 (0.075)	1.035 [0.982, 1.092]	−0.0004 (0.0009)
PD type (DSM-III system): *n* (%)							
Paranoid	26 (13.0)	20 (12.3)	51 (27.4)	30 (33.7)	24.53 (0.000)		
Schizoid	6 (3.0)	6 (3.7)	30 (16.1)	13 (14.6)	23.23 (0.000)		
Schizotypal	9 (4.5)	10 (6.2)	30 (16.1)	12 (13.5)	10.20 (0.001)		
** **Any Cluster A	**28 (14.0)**	**21 (13.0)**	**60 (32.3)**	**36 (40.4)**	**36.07 (0.000)**	1.047 [1.027, 1.067]***	−0.0030 (0.0011)**
Histrionic	27 (13.5)	9 (5.5)	22 (11.8)	6 (6.7)	8.54 (0.003)		
Antisocial	23 (11.5)	8 (4.9)	6 (3.2)	6 (6.7)	5.85 (0.016)		
Borderline	22 (11.0)	13 (8.0)	18 (9.7)	14 (15.7)	0.70 (0.404)		
Narcissistic	13 (6.5)	2 (1.2)	11 (5.9)	6 (6.7)	0.094 (0.759)		
Any Cluster B	**44 (22.0)**	**25 (15.3)**	**28 (15.1)**	**16 (18.0)**	**1.67 (0.197)**	0.985 [0.964, 1.006]	0.0018 (0.0012)
Avoidant	21 (10.5)	12 (7.4)	45 (24.2)	27 (30.3)	26.92 (0.000)		
Dependent	21 (10.5)	13 (8.0)	16 (8.6)	12 (13.5)	0.15 (0.696)		
Obsessive-compulsive	16 (8.0)	12 (7.4)	33 (17.7)	22 (24.7)	19.78 (0.000)		
Any Cluster C	**43 (21.5)**	**27 (16.7)**	**63 (33.9)**	**32 (36.0)**	**12.82 (0.000)**	1.031 [1.012, 1.050]**	−0.0019 (0.0010)
Passive-aggressive^b^	15 (7.5)	4 (2.5)	7 (3.8)	10 (11.2)	0.202 (0.653)		
** **Any PD	79 (39.5)	48 (29.6)	87 (46.8)	45 (50.6)	5.80 (0.016)	1.055 [1.003, 1.120]*	−0.0011 (0.0009)

PD: personality disorder; PAS: Personality Assessment Schedule; DSM: *Diagnostic and Statistical Manual of Mental Disorders*; AOR: adjusted odds ratio; CI: confidence interval; SE: standard error; DSM-III: *Diagnostic and Statistical Manual of Mental Disorders* (3rd ed.).

The set of patients analysed here was all patients with details shown in the Supplementary S2.

a Adjusted for age of patients.

b Passive-aggressive personality disorder is no longer diagnosed but was an established category in DSM-III.

* *p* < 0.05, **p < 0.01, ***p < 0.001.

### Changes in severity of personality status over follow-up

**Figure 1.**
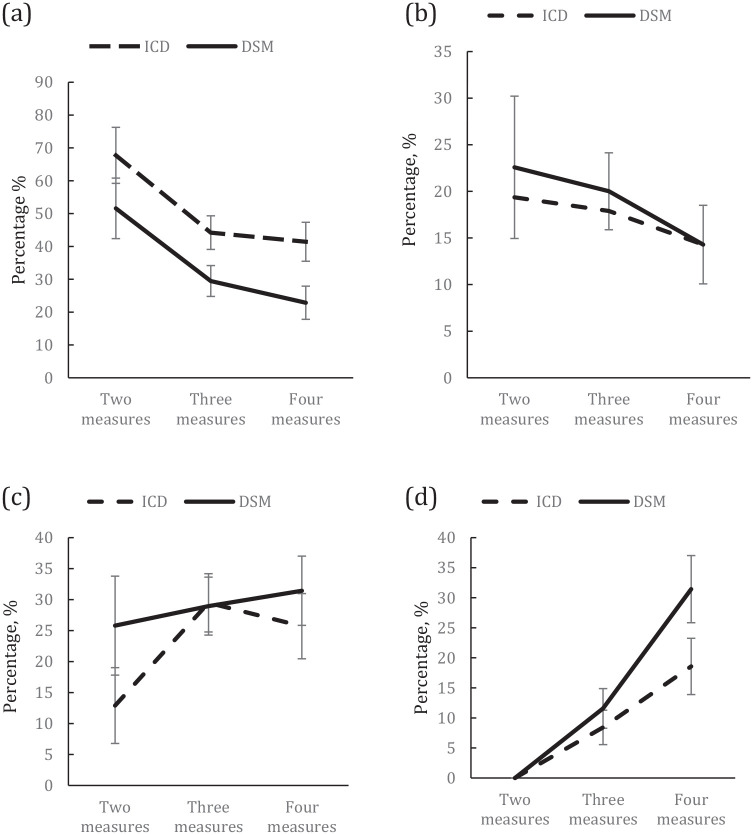
Comparison of change status between ICD and DSM personality disorder status in percentage and standard errors by number of measures over 30-year period: (a) patients who did not change, (b) patients who improved, (c) patients who became worse and (d) patients who oscillated in severity. The data show that most patients changed their personality status apart from those who had only two assessments.

There were many changes in the severity of personality disturbance over the follow-up period. Apart from 11 patients who had only one personality assessment over the study period, 92 (47%) had the same status, 33 (16.8%) improved status, 50 (20.4%) worsened status and 21 (10.7%) fluctuated from changes to worse from improved or vice versa over follow-up by the PAS PD system. The change in status by the DSM system was 30.6%, 18.4%, 34.2% and 16.8% (Table 2 and Figure 1). The distributions of changes between the two diagnostic systems were highly correlated (*r* = 0.796, *p* < 0.001), suggesting a similar change pattern in the two systems.

**Table 2.** Patient distribution by category of changes during follow-up period in personality disturbance defined by both PAS and DSM-III.

Category of PD change	Number of PD measures during follow-up time points *n* (%)			Total (%)
	Two	Three	Four	
PAS PD				
No change				
No PD all time points	19 (61.29)	42 (44.21)	28 (40.00)	89 (45.41)
Same PD all time points	2 (6.45)	0 (0.0)	1 (1.43)	3 (1.53)
Improved				
From any PD to no PD	5 (16.13)	11 (11.58)	8 (11.43)	24 (12.24)
From more PD to less PD types	1 (3.23)	6 (6.32)	2 (2.86)	9 (4.59)
Worsened				
From no PD to any one or more PD types	4 (12.90)	21 (22.11)	12 (17.14)	37 (18.88)
From less PD to more PD types	0 (0.0)	7 (7.37)	6 (8.57)	13 (6.63)
Fluctuated	0 (0.0)	8 (8.42)	13 (18.57)	21 (10.71)
DSM-III PD				
No change				
No PD all time points	15 (48.38)	25 (26.32)	14 (20.0)	54 (27.55)
Same PD all time points	1 (3.22)	3 (3.16)	2 (2.86)	6 (3.06)
Improved				
From any PD to no PD	5 (16.13)	14 (14.74)	8 (11.43)	27 (13.78)
From more PD to less PD types	2 (6.45)	5 (5.27)	2 (2.86)	9 (4.59)
Worsened				
From no PD to any one or more PD types	7 (22.58)	26 (27.37)	15 (21.43)	48 (24.49)
From less PD to more PD types	1 (3.22)	11 (11.58)	7 (10.0)	19 (9.69)
Fluctuated	0 (0.0)	11 (11.58)	22 (31.43)	33 (16.84)
Total	31 (100.0)	95 (100.0)	70 (100.0)	196 (100.0)

PD: personality disorder; PAS: Personality Assessment Schedule; DSM-III: *Diagnostic and Statistical Manual of Mental Disorders* (3rd ed.).

The set of patients analysed consists of all patients with details shown in the Supplementary S2.

Numbers are presented in Table 2.

These analysis excluded 11 patients whose PD change could not be observed because they had only one time PD measure.

### Changes in personality over time from baseline status

It was also considered important to know if baseline personality status was associated with changes over time. Personality difficulty is also included here as although it is not a personality disorder it is an important part of the personality spectrum (World Health Organization, 2018). The analysis showed a similar pattern of change over time as the previous analysis. Those with no personality dysfunction or with personality difficulty showed significant increases in frequency of all personality groups apart from sociopathic. Those with an existing personality disorder at baseline tended to remain disordered with only those with simple sociopathic disorder improving inconsistently (Table 3).

**Table 3.** ICD personality disturbance by personality status at baseline and changes over follow-up period using multilevel multivariate logistic models.

PD categories by severity level at baseline	Positive cases and percentage (%) at follow-up time points				Model estimated change parameter	
	Baseline	2 years	12 years	30 years	Linear change AOR [95% CI]^a^	Quadratic change Est (SE)
No PD: *n*	87	74	75	35		
Sociopathic	0	0	1 (1.3)	1 (2.9)	1.064 [0.978, 1.157]	N/A
Passive dependent	0	4 (5.4)	7 (9.3)	8 (22.9)	1.091 [1.041, 1.144]**	−0.0037 (0.0026)
Anankastic	0	0	5 (6.7)	2 (5.7)	1.071 [1.014, 1.131]*	N/A
Schizoid	0	2 (2.7)	7 (9.3)	2 (5.7)	1.069 [0.987, 1.158]	−0.0094 (0.0036)**
Any PD	0	5 (6.8)	18 (24.0)	10 (28.6)	1.131 [1.093, 1.171]***	−0.0098 (0.0017)***
PD difficulty: *n*	40	31	39	16		
Sociopathic	0	1 (3.2)	2 (5.1)	1 (6.3)	1.037 [0.954, 1.127]	N/A
Passive dependent	0	1 (3.2)	5 (12.8)	2 (12.5)	1.055 [0.995, 1.119]	N/A
Anankastic	0	1 (3.2)	4 (10.3)	3 (18.8)	1.079 [1.017, 1.146]*	N/A
Schizoid	0	0	5 (12.8)	3 (18.8)	1.101 [1.048, 1.156]**	N/A
Any PD	0	3(10.0)	10 (25.0)	4 (25.0)	1.109 [1.056, 1.166]**	−0.0086 (0.0024)**
Simple PD: *n*	51	41	47	27		
Sociopathic	19 (37.3)	4 (9.8)	4 (8.5)	4 (14.8)	0.969 [0.932, 1.007]	0.0071 (0.0030)*
Passive dependent	14 (27.5)	2 (4.9)	8 (17.0)	4 (14.8)	0.988 [0.949, 1.028]	0.0012 (0.0024)
Anankastic	9 (17.6)	6 (14.6)	9 (19.1)	5 (18.5)	1.004 [0.966, 1.044]	−0.0006 (0.0022)
Schizoid	4 (7.8)	3 (7.3)	10 (21.3)	5 (18.5)	1.033 [0.986, 1.081]	−0.0040 (0.0024)
Any PD	38 (74.5)	12 (29.3)	17 (36.2)	12 (44.4)	0.975 [0.947, 1.004]	0.0046 (0.0018)*
Complex PD: *n*	22	17	18	9		
Sociopathic	8 (36.4)	3 (17.6)	2 (11.1)	2 (22.3)	0.982 [0.927, 1.041]	0.0064 (0.0043)
Passive dependent	13 (59.1)	7 (41.2)	9 (50.0)	5 (55.6)	1.004 [0.962, 1.048]	0.0017 (0.0024)
Anankastic	12 (54.5)	6 (35.3)	9 (50.0)	7 (77.8)	1.044 [0.988, 1.103]	0.0024 (0.0029)
Schizoid	5 (22.7)	5 (29.4)	8 (44.4)	4 (44.4)	1.025 [0.978, 1.074]	−0.0025 (0.0026)
Any PD	21 (95.5)	12 (70.6)	14 (77.8)	7 (77.8)	0.970 [0.916, 1.026]	0.0037 (0.0030)

PD: personality disorder; ICD: International Classification of Diseases; AOR: adjusted odds ratio; CI: confidence interval; SE: standard error.

The set of patients analysed here for each severity level was 87, 40, 51 and 22, respectively.

a Adjusted for age of patients. N/A not estimated due to small sample size.

* *p* < 0.05, **p < 0.01, ***p < 0.001.

### Changes in DSM-III personality by baseline PAS severity

The DSM-III classification does not record severity of personality disturbance, but in the PAS system, the DSM-III personality disorders can be so classified. In Table 4, the changes in simple and complex DSM-III clusters of personality disorder showed that Cluster B personality disorders at the simple level of pathology (equivalent to mild personality disorder in the proposed ICD-11 classification) were the only group to show significant improvement over time; Clusters A and C appeared to worsen (Table 4). Those with complex personality disorder showed little change over time, which was similar to the group of PAS system in Table 3. The increased proportion of personality disturbance in all clusters over time was also consistent with those in the PAS system (Table 3).

**Table 4.** DSM Personality disturbance by personality status at baseline and changes over follow-up period using multilevel multivariate logistic models.

PD categories by severity level at baseline	Positive cases and percentage (%) at follow-up time points				Model estimated change parameter	
	Baseline	2 years	12 years	30 years	Linear change AOR [95% CI]^a^	Quadratic change Est (SE)
No PD: *n*	87	74	75	35		
Cluster A	0	3 (4.1)	12 (16.0)	8 (22.9)	1.172 [1.134, 1.212]***	−0.0092 (0.0015)***
Cluster B	0	7 (9.5)	8 (10.7)	6 (17.1)	1.091 [1.060, 1.123]***	−0.0052 (0.0015)**
Cluster C	0	6 (8.1)	20 (26.7)	12 (34.3)	1.186 [1.148, 1.225]***	−0.0104 (0.0015)***
Any PD	0	11 (14.9)	27 (36.0)	16 (45.7)	1.101 [1.069, 1.135]***	−0.0066 (0.0015)**
PD difficulty: *n*	40	31	39	16		
Cluster A	1 (2.5)	1 (3.2)	13 (33.3)	4 (25.0)	1.091 [1.029, 1.158]**	−0.0094 (0.0027)**
Cluster B	4 (10.0)	4 (12.9)	6 (15.4)	2 (12.5)	1.003 [0.952, 1.057]	N/A
Cluster C	8 (20.0)	3 (9.7)	15 (38.5)	7 (43.8)	1.047 [1.005, 1.092]*	−0.0028 (0.0022)
Any PD	13 (32.5)	6 (20.0)	20 (51.3)	7 (43.8)	1.024 [0.985, 1.064]	−0.0034 (0.0020)
Simple PD: *n*	51	41	47	27		
Cluster A	12 (23.5)	9 (22.0)	19 (40.4)	12 (44.4)	1.036 [1.004, 1.068]*	−0.0020 (0.0018)
Cluster B	29 (56.9)	9 (22.0)	6 (12.8)	3 (11.1)	0.925 [0.891, 0.959]***	0.0062 (0.0022)**
Cluster C	19 (37.3)	9 (22.0)	16 (34.0)	8 (29.6)	1.000 [0.970, 1.030]	−0.00015 (0.0017)
Any PD	44 (86.3)	18 (43.9)	23 (48.9)	14 (51.9)	0.965 [0.936, 0.994]*	0.0044 (0.0017)**
Complex PD: *n*	22	17	18	9		
Cluster A	15 (68.2)	7 (41.2)	13 (72.2)	8 (88.9)	1.056 [0.979, 1.138]	0.0012 (0.0034)
Cluster B	11 (50.0)	5 (29.4)	7 (38.9)	5 (55.6)	1.015 [0.964, 1.069]	0.0025 (0.0029)
Cluster C	16 (72.7)	9 (52.9)	12 (66.7)	5 (55.6)	0.987 [0.936, 1.040]	−0.0001 (0.0029)
Any PD	22 (100.0)	13 (76.5)	15 (83.3)	8 (88.9)	0.991 [0.928, 1.059]	0.0051 (0.0032)

PD: personality disorder; DSM: *Diagnostic and Statistical Manual of Mental Disorders*; AOR: adjusted odds ratio; CI: confidence interval; SE: standard error.

The set of patients analysed here for each severity level was 87, 40, 51 and 22, respectively.

a Adjusted for age of patients. N/A not estimated due to small sample size.

* *p* < 0.05, **p < 0.01, ***p < 0.001.

### Discussion

There are three findings from this study that both challenge and support current notions of the course of personality disorder. The first is that personality disorder is unstable over time, the second that, rather than attenuating with age, the tendency is for some personality disturbance to become more pronounced (independent of organic change), and the third is that the form or type of personality disturbance changes over time to different degrees.

To some degree, these conclusions are supported by the existing literature. Longitudinal studies have shown considerable variation in personality status over time (Skodol, 2008; Warner et al., 2004; Zanarini et al., 2003), but most studies have not made formal personality assessments repeatedly. There have been many reports of improvement to no personality disorder over time; Skodol (2008) describes this as ‘personality psychopathology improves over time at unexpectedly significant rates’, but the populations concerned involved many specifically selected for their personality disorders and often receiving long-term treatment. There are also no studies that have examined change of a period as long as 30 years. The Nottingham study did not involve highly selected patients, had much longer follow-up and did not include specific treatment for personality disorder, so is better placed to report the natural history of personality disturbance over time.

The shift in type of personality disturbance over time, with reduction of some, particularly linked to aggressive characteristics in the Cluster B personality group, but increase in personality disorders in the Cluster A and C groups, is given support in the literature (Gunderson et al., 2011; Reichborn-Kjennerud et al., 2015; Zanarini et al., 2017) and illustrates the more ephemeral quality of categories as opposed to dimensions of personality disturbance (Morey et al., 2012).

The results also show that the absence of a personality disorder at baseline does not protect individuals from getting personality disturbance in all areas of personality later in life, particularly in the longer term. The new ICD-11 classification allows the diagnosis of personality disorder to be made at any age provided that the features satisfying the diagnosis have been present for 2 years or more (Tyrer et al., 2019; World Health Organization, 2018). Little is known about late-onset personality disorder, but there is general agreement among psychogeriatricians that such disturbance is a clinical entity and is particularly manifest after bereavement and care home placement (Rosowsky et al., 2019).

The challenging finding of this study is that, with the exception of some Cluster B personalities, most people with personality disorder do not improve over a long time period. Deterioration is rarely a linear course and the greater significance of quadratic change confirms this. Most studies suggesting improvement have been over much shorter times. The exception is the longitudinal cohort of Zanarini et al. (2003, 2006, 2007) which found that 88% of all patients with borderline personality disorder had remitted by 10 years and similar improvement was found in a subsample of other (unspecified) personality disorders. The patients in the Zanarini studies, all of whom were inpatients initially, also continued to receive therapy, with both drugs and psychotherapy, and the authors argue that ‘therapy is essential to lay the ground-work for a better, less painful life’ (but in the absence of a control group this cannot be assumed). In some cases, therapy even turns into a life-style and is the most important element in a patient’s life. We share the belief that therapy can and often does encourage and facilitate change (Zanarini, 2008).

The findings of the Zanarini study also show the dramatic improvement of those with borderline personality disorder in her study in a different light. The patients originally diagnosed with a Cluster B personality disorder in the Nottingham study were the only ones to lose personality disorder status over time (Table 4); all others, including those with little or no personality disturbance at baseline, showed varying degrees of worsening pathology. So those with Cluster B pathology were different from other clusters in the spectrum of personality disturbance.

None of the patients in the Nottingham Study received any specific treatment for personality disorder and this might account for some of the differences in outcome in other studies. Nonetheless, in the Zanarini studies, the symptoms that were most resistant to improvement were chronic anxiety and depression (Zanarini et al., 2007, 2019), suggesting that those in the NSND population with personality disorder were not very different from the Zanarini population.

Long-term follow-up studies are becoming increasingly difficult to carry out but the 30-year data has shed additional light on personality development that needs to be replicated, particularly in view of the increasing proportion of elderly people in the community.

## Conclusion

The data presented here allow firm conclusions. Personality disorder should no longer be considered to be a dichotomous entity separating it from no personality disorder. The marked fluctuations in the presentation of both the severity and domains of disorder over 30 years show that it is preferable to view personality across the life course as a spectrum of pathology, where many changes can occur. Understanding the nature and reasons for these, especially the tendency for pathology in general to increase over time, is a key area of future enquiry. These findings suggest it is much better to regard a single assessment of personality status as one of present personality function than one of disorder (Clark, 2005; Tyrer et al., 2007). Changes in status in this population of those who had anxiety and depressive disorders at baseline may not necessarily be representative of the population as a whole, but they do suggest that formal definition of personality disorder should no longer include an indication of long-term stability. The reasons for instability are outside the realm of this paper but are likely to include changes in mental state, the effect of age and environmental factors (Caspi et al., 2005; Coppen and Metcalfe, 1965; Tyrer, 2002). The findings also suggest that much more attention needs to be given to the assessment and management of personality pathology than is currently the case.

## Supplemental Material

sj-docx-1-anp-10.1177_00048674211025624 – Supplemental material for Personality change in the Nottingham Study of Neurotic Disorder: 30-Year cohort study Click here for additional data file. Supplemental material, sj-docx-1-anp-10.1177_00048674211025624 for Personality change in the Nottingham Study of Neurotic Disorder: 30-Year cohort study by Min Yang, Helen Tyrer, Tony Johnson and Peter Tyrer in Australian & New Zealand Journal of Psychiatry
