# Supplementary material for: Personality change in the Nottingham Study of Neurotic Disorder: 30-Year cohort study
Source: Aust N Z J Psychiatry. 2021 Jul 10;56(3):260–9. doi: 10.1177/00048674211025624 (PMC8866742; doi:10.1177/00048674211025624)
Supplement: sj-docx-1-anp-10.1177_00048674211025624 – Supplemental material for Personality change in the Nottingham Study of Neurotic Disorder: 30-Year cohort study [file sj-docx-1-anp-10.1177_00048674211025624.docx]

Supplementary Table S1: Baseline characteristics of individuals who had died or were lost to follow-up

| Follow-up point |  | Assessed | Lost follow-up | Dead (cumulative) | P value^a^ | P value^b^ |
| --- | --- | --- | --- | --- | --- | --- |
| 2 years | **N** | **165** | **44** | **1** |  |  |
|  | Age at allocation: mean(SD) | 37.8 (12.2) | 31.0 (12.1) | 28.0 (N/A) | **0.004** | **0.016** |
|  | Female: n (%) | 112 (67.9) | 32 (72.7) | N/A | 0.715 | 0.959 |
|  | Marital status: n (%)  Married  Separated  Divorced  Widowed  Never married | 72 (43.6)  9 (5.5)  31 (18.8)  4 (2.4)  49 (29.7) | 10 (22.7)  4 (9.1)  7 (15.9)  1 (2.3)  22 (50.0) | 1  0  0  0  0 | 0.072 | 0.452 |
|  | Social class: n (%)  Professional  Intermediate  Skilled manual/non manual  Semi-skilled manual/non manual  Non-skilled manual/non manual | 7 (4.2)  14 (8.5)  36 (21.8)  69 (41.8)  39 (23.6) | 2 (4.5)  5 (11.4)  9 (20.5)  7 (15.9)  21 (47.7) | 0  0  0  1  0 | **0.008** | 0.354 |
|  | Treatment group: n (%)  Drug  CBT  Self-help | 65 (39.4)  65 (39.4)  35 (21.2) | 19 (43.2)  18 (40.9)  7 (15.9) | 0  1  0 | 0.842 | Not included |
|  |  |  |  |  |  |  |
|  | DSM III diagnoses: n (%)  DYS  GAD  Panic  Cothymia | 5 (3.0)  52 (31.5)  52 (31.5)  56 (33.9) | 4 (9.1)  18 (40.9)  7 (15.9)  15 (34.1) | 0  0  1  0 | 0.070 | 0.176 |
| 12 years | **N** | **185** | **8** | **17** |  |  |
|  | Age at allocation: mean(SD) | 35.4 (11.5) | 34.4 (18.6) | 47.8 (14.4) | **0.000** | **0.004** |
|  | Female: n (%) | 126 (68.1) | 4 (50.0) | 15 (88.2) | 0.107 | 0.249 |
|  | Marital status: n (%)  Married  Separated  Divorced  Widowed  Never married | 74 (40.0)  11 (5.9)  35 (18.9)  3 (1.6)  62 (33.5) | 0 (0.0)  1 (12.5)  1 (12.5)  0 (0.0)  6 (75.0) | 9 (52.9)  1 (5.9)  2 (11.8)  2 (11.8)  3 (17.7) | **0.021** | 0.158 |
|  | Social class: n (%)  Professional  Intermediate  Skilled manual/non manual  Semi-skilled manual/non manual  Non-skilled manual/non manual | 7 (3.8)  19 (10.3)  40 (21.6)  67 (36.2)  52 (28.1) | 2 (25.0)  0 (0.0)  2 (25.0)  2 (25.0)  2 (25.0) | 0 (0.0)  0 (0.0)  3 (17.6)  8 (47.1)  6 (35.3) | 0.327 | 0.204 |
|  | Treatment group: n (%)  Drug  CBT  Self-help | 74 (40.0)  74 (40.0)  37 (20.0) | 5 (62.5)  3 (37.5)  0 (0.0) | 5 (29.4)  7 41.2)  5 (29.4) | 0.195 | Not included |
|  | GNS status (score ≥ 6) | 64 (35.0) | 0 (0.0) | 7 (43.8) | 0.155 | Not included |
|  | DSM III diagnoses: n (%)  DYS  GAD  Panic  Cothymia | 7 (3.8)  62 (33.5)  54 (29.2)  62 (33.5) | 1 (12.5)  3 (37.5)  1 (12.5)  3 (37.5) | 1 (5.9)  5 (29.4)  5 (29.4)  6 (35.3) | 0.726 | Not included |
| 30 years | **N** | **87-88** | **51-54** | **67-68** |  |  |
|  | Age at allocation: mean(SD) | 30.7 (8.6) | 32.6 (10.6) | 46.6 (11.7) | **0.000** | **0.000** |
|  | Female: n (%) | 63 (71.6) | 38 (70.4) | 44 (64.7) | 0.661 | 0.952 |
|  | Marital status: n (%)  Married  Separated  Divorced  Widowed  Never married | 30 (34.1)  8 (9.1)  13 (14.8)  0 (0.0)  37 (42.0) | 22 (40.7)  2 (3.7)  10 (18.5)  0 (0.0)  20 (37.0) | 31 (45.6)  3 (4.4)  15 (22.1)  5 (7.4)  14 (20.6) | **0.015** | 0.897 |
|  | Social class: n (%)  Professional  Intermediate  Skilled manual/non manual  Semi-skilled manual/non manual  Non-skilled manual/non manual | 7 (8.0)  11 (12.5)  16 (18.2)  30 (34.1)  24 (27.3) | 2 (3.7)  4 (7.4)  17 (31.5)  15 (27.8)  16 (29.6) | 0 (0.0)  4 (5.9)  12 (17.6)  32 (47.1)  20 (29.4) | 0.070 | 0.082 |
|  | Treatment group: n (%)  Drug  CBT  Self-help | 32 (36.4)  39 (44.3)  17 (19.3) | 25 (46.3)  18 (33.3)  10 (18.5) | 26 (38.2)  27 (39.7)  15 (22.1) | 0.182 | Not included |
|  | GNS status (score ≥ 6) | 28 (32.2) | 18 (35.3) | 25 (37.3) | 0.789 | Not included |
|  | DSM III diagnoses: n (%)  DYS  GAD  Panic  Cothymia | 1 (1.1)  26 (29.5)  32 (36.4)  29 (33.0) | 4 (7.4)  20 (37.0)  9 (16.7)  21 (38.9) | 4 (5.9)  24 (35.3)  19 (27.9)  21 (30.9) | 0.115 | Not included |

^a^Fisher’s exact test for categorical variables and ANOVA for mean age at randomization. ^b^Likelihood ratio test from fitting nominal multinomial model for 12- and 30-years follow-up groups, and Ward test from fitting logistic regression model for 2-year follow-up group.

Supplementary Table S2: Patten of any PD change (1 positive, 0 negative)

| 10 wks | 2 years | 12 years | 30 years | ICD PD: n (%) | DSM PD: n (%) |
| --- | --- | --- | --- | --- | --- |
| One time point: **11 cases** | | | | | |
| Two follow-up points | | | | | |
| 0 | 0 | -- | -- | 8 (25.8) | 6 (19.4) |
| 0 | -- | 0 | -- | 10 (32.3) | 8 (25.8) |
| -- | -- | 0 | 0 | 1 (3.2) | 1 (3.2) |
| 1 | 1 | -- | -- | 1 (3.2) | 2 (6.5) |
| 1 | -- | 1 | -- | 2 (6.5) | 2 (6.5) |
| 1 | 0 | -- | -- | 4 (12.9) | 3 (9.7) |
| 1 | -- | 0 | -- | 1 (3.2) | 1 (3.2) |
| -- | -- | 1 | 0 | 0 (0) | 1 (3.2) |
| 0 | 1 | -- | -- | 2 (6.5) | 4 (12.9) |
| 0 | -- | 1 | -- | 1 (3.2) | 3 (9.7) |
| -- | -- | 0 | 1 | 1 93.2) | 0 (0.0) |
| **Subtotal** |  |  |  | **31 (100.0)** | **31 (100.0)** |
| Three follow-up points | | | | | |
| 0 | 0 | 0 | -- | 39 (41.1) | 24 (25.3) |
| 0 | -- | 0 | 0 | 4 (4.2) | 0 (0.0) |
| 1 | 1 | 1 | -- | 6 (6.3) | 14 (14.7) |
| 1 | -- | 1 | 1 | 2 (2.1) | 0 (0.0) |
| 1 | 1 | 0 | -- | 2 (2.1) | 3 (3.2) |
| 1 | 0 | 1 | -- | 4 (4.2) | 4 (4.2) |
| 0 | 1 | 1 | -- | 3 (3.2) | 2 (2.1) |
| 1 | 1 | 0 | -- | 0 (0.0) | 3 (3.2) |
| 0 | -- | 1 | 1 | 2 (2.1) | 3 (3.2) |
| 1 | -- | 0 | 1 | 3 (3.2) | 2 (2.1) |
| 0 | 1 | 0 | -- | 4 (4.2) | 6 (6.3) |
| 0 | 0 | 1 | -- | 13 (13.7) | 19 (20.0) |
| 1 | 0 | 0 | -- | 6 (6.3) | 8 (8.4) |
| 1 | -- | 0 | 0 | 3 (3.2) | 3 (3.2) |
| 0 | -- | 1 | 0 | 2 (2.1) | 2 (2.1) |
| 0 | -- | 0 | 1 | 2 (2.1) | 2 (2.1) |
| **Subtotal** |  |  |  | **95 (100.0)** | **95 (100.0)** |
| Four follow-up points | | | | | |
| 0 | 0 | 0 | 0 | 28 (40.0) | 12 (17.1) |
| 1 | 1 | 1 | 1 | 5 (7.1) | 9 (12.9) |
| 1 | 1 | 0 | 1 | 1 (1.4) | 2 (2.9) |
| 1 | 0 | 1 | 1 | 5 (7.1) | 5 (7.1) |
| 0 | 1 | 1 | 1 | 1 (1.4) | 1 (1.4) |
| 1 | 1 | 1 | 0 | 2 (2.9) | 2 (2.9) |
| 0 | 0 | 1 | 1 | 4 (5.7) | 6 (8.6) |
| 1 | 1 | 0 | 0 | 2 (2.9) | 2 (2.9) |
| 1 | 0 | 0 | 1 | 1 (1.4) | 2 (2.9) |
| 1 | 0 | 1 | 0 | 1 (1.4) | 2 (2.9) |
| 0 | 1 | 1 | 0 | 1 (1.4) | 1 (1.4) |
| 0 | 0 | 0 | 1 | 7 (10.0) | 10 (14.3) |
| 1 | 0 | 0 | 0 | 5 (7.1) | 6 (8.6) |
| 0 | 0 | 1 | 0 | 5 (7.1) | 7 (10.0) |
| 0 | 1 | 0 | 0 | 2 (2.9) | 3 (4.3) |
| **Subtotal** |  |  |  | **70 (100.0)** | **70 (100.0)** |
| Total |  |  |  | 207 | 207 |
